# Cardiovascular Risk Factors in Patients with Congenital Hemophilia: A Focus on Hypertension

**DOI:** 10.3390/diagnostics12122937

**Published:** 2022-11-24

**Authors:** Minerva Codruta Badescu, Oana Viola Badulescu, Lăcrămioara Ionela Butnariu, Iris Bararu Bojan, Maria Cristina Vladeanu, Nicoleta Dima, Cristiana Elena Vlad, Liliana Georgeta Foia, Manuela Ciocoiu, Ciprian Rezus

**Affiliations:** 1 Department of Internal Medicine, “Grigore T. Popa” University of Medicine and Pharmacy, 16 University Street, 700115 Iasi, Romania; 2 III Internal Medicine Clinic, “St. Spiridon” County Emergency Clinical Hospital, 1 Independence Boulevard, 700111 Iasi, Romania; 3 Department of Pathophysiology, “Grigore T. Popa” University of Medicine and Pharmacy, 16 University Street, 700115 Iasi, Romania; 4 Hematology Clinic, “St. Spiridon” County Emergency Clinical Hospital, 1 Independence Boulevard, 700111 Iasi, Romania; 5 Department of Mother and Child Medicine, “Grigore T. Popa” University of Medicine and Pharmacy, 700115 Iasi, Romania; 6 “Dr. C. I. Parhon” Clinical Hospital, 50 Carol I Boulevard, 700503 Iasi, Romania; 7 Department of Biochemistry, “Grigore T. Popa” University of Medicine and Pharmacy, 16 University Street, 700115 Iasi, Romania

**Keywords:** hemophilia, hypertension, cardiovascular risk, cardiovascular disease, kidney dysfunction

## Abstract

Aging hemophiliacs face cardiovascular disease. Lots of evidence has been gathered that hemophiliacs have a more unfavorable cardiovascular profile than the general population does, especially due to the increased prevalence of hypertension (HTN). Among the existing scattered evidence, our study provides the most comprehensive and systematized analysis of the determinants of HTN in hemophiliacs. We discussed the contribution to the HTN substrate of hemophilia-specific factors, such as type, severity and the presence of inhibitors. The complex mechanism of kidney dysfunction in relation to hematuria and viral infections was meticulously addressed. Furthermore, we highlighted the new pathogenic concepts of endothelial dysfunction and the association between HTN and hemophilic arthropathy. The clustering of cardiovascular risk factors is common in hemophiliacs, and it enhances the negative vascular effect of HTN and aggravates HTN. It usually leads to an increased risk for coronary and cerebrovascular events. Our work provides reliable evidence to guide and improve the management of HTN in hemophiliacs.

## 1. Introduction

Hemophilia is an X-linked genetic disease with a clinical expression in men. There are 17.1 cases of hemophilia A (factor VIII deficiency) per 100,000 men, which makes it the most frequent congenital bleeding disorder [1]. However, the prevalence of hemophilia B (factor IX deficiency) is 4.5 times lower, at only 3.8 cases per 100,000 men. The wide availability and implementation of substitution therapies have determined an increase in the life expectancy of hemophiliacs, which is currently similar to that of the general male population. The increase in the longevity of hemophiliacs has created the conditions for the accumulation of various health conditions, including cardiovascular ones.

Atherosclerosis affects all of the arterial territories, and it has been proven that the deficiency of coagulation factors does not have a protective role against its occurrence and progression [2]. Thus, coronary, cerebrovascular, and peripheral arterial diseases have begun to have an increasing prevalence. Coronary artery disease is quite common in hemophiliacs, and it can be encountered in all forms, from asymptomatic atherosclerosis to chronic coronary syndromes and acute myocardial infarction [3,4]. Although they are rarer than acute coronary syndromes, cerebral ischemic events represent a considerable concern due to their high mortality and major disabling character [5]. Reports of peripheral artery disease (PAD) are rare as well. This may be due to the fact that claudication, the most characteristic clinical sign of PAD of the lower limbs, is erroneously attributed to hemophilic arthropathy, is masked by analgesics which are used to treat joints pain or is absent due to the severe arthropathy that does not allow enough mobility [4]. The association of rare comorbidities can further hamper the diagnosis. On extremely rare occasions, the coexistence of hemophilia and sickle cell disease has been reported [6]. Sickle cell disease is an inherited blood disorder that manifests as painful vaso-occlusive crises. Muscular pain and damage to the large joints are among its manifestations. Moreover, autoimmune diseases affecting the joints and muscles can overlap, and this makes it difficult to identify the origin of the pain [7,8].

Considering the need for antithrombotic therapy arising from the presence of atherosclerotic vascular diseases, managing these patients has proved to be challenging on account of the innate and high hemorrhagic risk. In this context, its prevention was given more thought in an attempt to avoid or at least postpone the onset of cardiovascular disease and its specific treatment with a bleeding potential. Since the coronary, cerebral and peripheral arteries diseases have a commune substrate—atherosclerosis—and they share the same risk factors, early interventions could lower cardiovascular morbidity and mortality.

The cardiovascular risk factors identified in the general population were sought in hemophiliacs as well. Their correction is considered to be the best method of intervention for cardiovascular diseases in this particular population category. There are constant reports that hypertension (HTN) is more prevalent in hemophiliacs than it is in the general population. Because in the long term its evolution is linked to numerous complications, such as stroke, myocardial infarction and chronic kidney disease, all of which have a significant clinical, therapeutic and prognostic impact in hemophiliacs, understanding the particularities of the etiological substrate in this population is of great importance. Congenital hemophilia is a condition with low prevalence [1], therefore, the studies of this have generally included a small number of patients, which represents an impediment in generating an overall picture with respect to the HTN substrate. Our work aims to fill this gap, providing the most up-to-date, systematized and comprehensive analysis of HTN in individuals with congenital hemophilia.

## 2. Hypertension

The first reports on the increased prevalence of HTN in hemophiliacs are from the early 1990s. Both the systolic and diastolic blood pressure values were higher among the hemophiliacs than they were in the general population and for the use of antihypertensive treatments as well [9]. The prevalence of HTN was 1.9 times higher in the hemophiliacs than it was in the controls, and it was strongly associated with age. As much as 58% of the hypertensives were over 50 years old. Given that the blood pressure (BP) values from which HTN is currently defined are lower than those that were used at the time of this study, it was assumed that the prevalence of the disease is much higher.

Robust data are provided by a large North-European study [10]. The prevalence of HTN was higher in the hemophiliacs than it was in the general population of men, and this was strongly correlated with age, with it being 24% of the hemophiliacs aged 30–40 years and as high as 82% in those over 60. An antihypertensive treatment was identified in 25% of the hemophiliacs, but worryingly, 57% of them were undertreated. Similar results were published by Holme et al. In their study, the prevalence of HTN increased with age from 23.7% in those aged 40–50 years to 79.7% in those over 70 years. [11]. One German and two Italian studies conducted on geriatric hemophiliac populations found a prevalence of HTN that was equal to or higher than that in individuals of the same sex and age in the general population [12,13,14].

A high prevalence of HTN in hemophiliacs when they were compared to the general population has also been detected in North American studies [15,16,17]. It was clearly noted that hemophiliacs become hypertensive at a young age. In the US, the hemophiliacs aged 18–44 years had a prevalence of NTH that was 2.5 times higher than it was in the age-matched men in the general population (31.8% vs. 12.5%) [15]. Pocoski et al. study noted that the difference between the prevalence of it starts early in life, at the age of 30 [17]. It has also been observed that the prevalence of HTN in hemophiliacs increases progressively with age, and it remains higher than that of the general population across all of the age groups. Even after adjusting for human immunodeficiency virus (HIV) and hepatitis C virus (HCV), the difference in the prevalence remained significant—20.5% of the hemophiliacs vs. 14% of the general population. The ARCHER study, which included the largest Canadian cohort of hemophiliacs, confirmed the previous results [18]. There is only one study reporting HTN in South-American hemophiliacs [19]. It showed that the prevalence of HTN is higher in hemophiliacs than it is in the general population of Pernambuco and Brazil. Asian studies generally report a lower prevalence of HTN than the European and American ones did [20,21,22,23]. The lowest prevalence was 10.63% and the highest was 43.7%, and in both cases, it was similar to that in the general population [22,23].

The increase in the prevalence of HTN in the hemophiliacs, starting at the age of 30, which is at least a decade earlier than in the general population, was noted in several studies across the continents [10,11,15,17]. One study showed that half of the participants had an elevated BP or HTN [24]. Recently, the accumulated evidence has shown that altered BP values are present even in the youngest hemophiliacs. Of forty-three patients aged 5–20 years, with only six adults being included, 28% of them had an elevated BP or HTN [25]. Moreover, there were reports that the prevalence of HTN in children was higher in the hemophiliacs than it was in the general population [26].

### 2.1. Hemophilia Type

**Figure 1:**
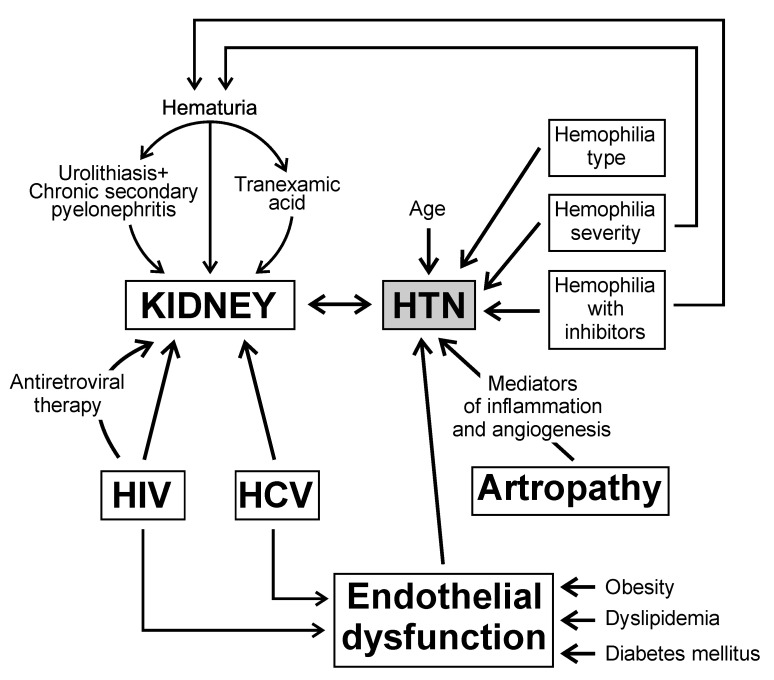
The mechanisms of hypertension in patients with congenital hemophilia.

The data available for comparison are limited because the disease is rare, and hemophilia A (HA) is much more widespread than hemophilia B is (HB). Moreover, the available results are very different, so it is difficult to draw a conclusion. Von Drygalski et al. found that patients with HB had 1.87 times higher risk of being hypertensive when they were compared to those with HA [15] (Figure 1). This finding contradicts the results of the study by Ter-Zakarian et al. who observed a higher prevalence of hypertension and a higher BP in HA patients when they were compared to HB patients [27]. In general, the studies have not found an association between the type of hemophilia and the prevalence of HTN [10,11].

### 2.2. Hemophilia Severity

An association between the severity of hemophilia and the prevalence of HTN has been investigated in several studies. Many reports show that the prevalence of HTN is similar across hemophilia severities, and the authors consider that there is no association between the severity of hemophilia and the prevalence of HTN [9,28,29,30]. However, in a large cohort of hemophiliacs who were older than 40, a slightly higher prevalence of HTN was identified in the patients with severe hemophilia (18.2%) when they were compared to those with a non-severe disease (15.1%) [31]. Another study found that hemophiliacs with a moderate or severe disease had a 1.3-fold increased risk of having HTN when they were compared with those with a mild disease [15]. Since hematuria is common in hemophiliacs, especially in those with a severe disease, and given the bidirectional relationship between HTN and renal disease, it was hypothesized that renal bleeding might be involved in the pathogenesis of HTN. The recent and opposite results of Ter-Zakarian et al., namely that there are more HTN cases in moderate hemophilia groups than there are in severe hemophilia groups, show that this issue needs further investigation [27].

### 2.3. Hemophilia with Inhibitors

Inhibitors are usually found in patients with severe hemophilia that require a coagulation factor replacement therapy, and they appear early in life, during childhood. Patients with mild hemophilia rarely require replacement therapy, but nowadays, with the increase in the life span of hemophiliacs, the spectrum of medical or surgical conditions that they develop and which require the administration of coagulation factors has increased [32]. This is a significant issue as the presence of inhibitors increases the hemorrhagic risk and makes the response to replacement therapy unpredictable.

Since the patients with inhibitors are more prone to bleeding compared to hemophiliacs without inhibitors, the hypothesis of repeated microbleeds in the kidney leading, over time, to renal failure was considered as a substrate for HTN. Moreover, the inhibitors have the potential to influence vascular health through an immune complex formation.

A small Japanese study reported that the prevalence of HTN showed a strong relationship with the presence of inhibitors [23]. Of 29 patients with inhibitors, 23 (79.3%) patients had HTN. However, one large study, enrolling 691 North-American hemophiliacs, of which 6.5% of them had inhibitors, did not report a higher prevalence of HTN or higher BP in hemophiliacs with inhibitors when they were compared to those without inhibitors [27].

### 2.4. Role of the Kidney

**Figure 2:**
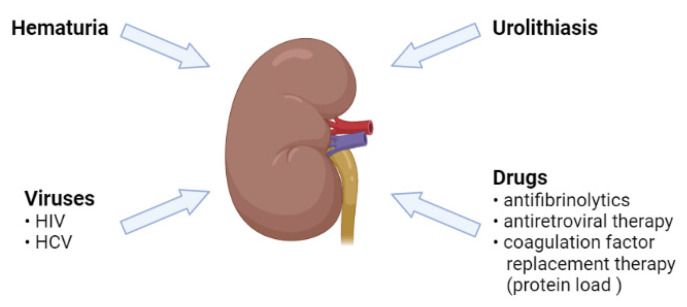
The major determinants of renal dysfunction in patients with congenital hemophilia.

Similar to the general population, HTN in hemophiliacs was independently associated with renal function, which was assessed by both the creatinine and estimated glomerular filtration rate (eGFR) [15]. Hematuria is common in hemophiliacs, and it was assumed that in the long term it could possibly negatively influence the renal function and lead to HTN [33]. HTN was 1.75 times more prevalent in the individuals with severe hemophilia than in those with moderate and mild diseases and although the renal function was normal in 98% of hemophiliacs, 33% of them had a history of renal bleeding [10]. There were also isolated case reports linking the use of tranexamic acid to kidney damage. Tranexamic acid is an antifibrinolytic agent, and when it is used for hematuria, it may determine clot formation and acute renal cortical necrosis [34,35]. Furthermore, hemophiliacs have a higher risk of HCV and HIV infection, and as a result of both viral infection and nephrotoxicity to an antiviral treatment, they may have a higher risk of kidney dysfunction. In this complex setting, it is expected that kidney diseases will have an increased prevalence, and due to the kidney–BP relationship, contribute to HTN (Figure 2).

An analysis of a North-American database of hospitalized hemophiliacs showed that HTN was strongly associated with both acute and chronic kidney diseases, and it suggested that a connection between the kidney bleeds and HTN might be possible. The prevalence of HTN was 10.1% in the hemophiliacs who were hospitalized for kidney bleeds and only in 4.5% of those who were admitted for other indications [33]. Acute renal disease was associated with HIV infection, HTN and the presence of inhibitors, while the chronic renal disease was mainly linked to HIV infection, HTN, kidney bleeds and diabetes.

The relationship between chronic hematuria, kidney dysfunction and HTN was the main focus of the H3 (Hypertension and Hematuria in Hemophilia) study, which enrolled 532 patients aged over 40 [11]. It has been hypothesized that renal bleeding—with hematuria as a marker—leads to renal dysfunction, which further leads to HTN. Although 58.9% of the hemophiliacs had a severe disease and 51.5% of them had past episodes of hematuria, the renal dysfunction was low in this cohort (5.5%). The severity of hemophilia and the history of hematuria did not correlate significantly with HTN. Surprisingly and unexpectedly, the mean eGFR was higher in the hemophiliacs with a severe disease than in those with a mild disease [11]. Still, an eGFR < 70 mL/min was significantly associated with HTN. A further analysis of this population found that the prevalence of HTN increased with the prevalence of macroscopic hematuria only in the hemophiliacs with a family history of HTN [36]. This led to the conclusion that hematuria acts as an enhancer and contributes to the transformation of predisposition into disease. Moreover, an increase in the risk of HTN was noted along with the increase in the number of episodes of macroscopic hematuria. Given that the severity of hematuria is correlated with the severity of hemophilia, attention was drawn towards the implications of prophylaxis. Qvigstad et al. highlighted the benefits of frequent long-standing prophylaxis over on-demand prophylaxis in reducing the number of episodes of macroscopic hematuria [37].

Another issue is whether a microscopic hematuria is as important as a macroscopic hematuria in the occurrence of HTN. Since one in three hemophiliacs has a microscopic hematuria, this issue raised concern. The recent data suggest that a microscopic hematuria is not a risk factor for either renal dysfunction or HTN [28].

A low prevalence of chronic kidney disease (CKD) in hemophiliacs was found in several studies. In a cohort of one hundred and thirty-five hemophiliacs, 34% of them had hematuria and 44% of them had HTN, but only three patients had CKD [28]. These results are valuable because unlike other studies that relied only on the history of gross hematuria, this study also included screening for asymptomatic hematuria by urinalysis/microscopy. Similarly, in a cohort of 82 hemophiliacs, only one case of CKD was identified [19].

It was shown that even in the youngest hemophiliacs with normal serum creatinine values, there are frequent structural and functional renal abnormalities and HTN. In a group of 40 children with severe and moderate HA, renal scintigraphy revealed obstructive uropathy and obstructive nephropathy in 35% and 37.5% of the patients, respectively [38]. The diminished GFR was found in 47.5% of the patients, but it was without clinical importance. A low prevalence of hematuria (14/40 cases) and hypertension (5/40 cases), and a diminished GFR appear to be linked, but a direct causality between hematuria and renal disease could not be established [38].

A study on 93 hemophiliacs found a prevalence of HTN in 27.9% of them, and urolithiasis and chronic secondary pyelonephritis in 21.5% of them [39]. When only hemophiliacs over 45 years were assessed, the prevalence of HTN raised to 64%. Although the relationship between HTN and renal dysfunction was not assessed, it is worth noting that the prevalence of urolithiasis and chronic secondary pyelonephritis also increased in those over 45 years. Hematuria is an important contributor to urolithiasis, and it was shown that hemophiliacs have a twice-increased risk of developing urolithiasis during their lifetime when they were compared to the general population [40]. Although a direct relationship between urolithiasis and HTN has not been demonstrated, it must be taken into account that urolithiasis is a contributor to the occurrence of CKD. In a small Asian study, HTN and nephrolithiasis were the most prevalent diseases in the hemophiliacs, with them occurring in 19% of the population, each [20].

Additionally, last but not least, the impact of protein load due to frequent coagulation factor replacement therapy should be considered, at least theoretically, as a contributor to renal failure and HTN [31,41].

### 2.5. Hemophilic Arthropathy

A new and interesting concept is that hemophilic arthropathy and increased BP are connected [42]. Vascular remodeling in HTN is a very complex process [43]. Systemic and local triggers, such as mechanical stress, bioactive peptides and reactive oxygen-nitrogen species, induce the activation of matrix metalloproteinases (MMPs). They are mainly responsible for extracellular matrix (ECM) degradation, a process that allows vascular smooth muscle cells (VSMCs) to detach from the matrix to migrate and proliferate. It also allows the inflammatory cells to cross the endothelium and infiltrate the vascular wall. Increased activity of MMP-2 was identified in both the early and adaptive phases of HTN and in the chronic and maladaptive phases. MMP-2 not only has proteolytic effects on the ECM constituents, but it also activates intracellular signaling pathways that determine the change in VSMCs phenotype from contractile to secretory. This allows the VSMCs to migrate and synthesize newer ECM components. Since MMP-2 is more abundant in secretory VSMCs than it is in contractile VSMCs, the synthesis process is intensified by changing the phenotype. Moreover, MMP-2 activates local cytokine synthesis, which further encourages the VSMC phenotype to switch and migrate. These changes in the vascular wall are accompanied by increased collagen deposition, elastin fragmentation and fibrosis, which all lead to arterial stiffness and sustained HTN.

In experimental murine studies, hemophilic arthropathy was characterized by excessive soft tissue proliferation, intensive neoangiogenesis and abnormal vascular architecture [44]. The vascular remodeling in intraarticular soft tissue was reminiscent of the vascular remodeling in HTN. Hemarthrosis in hemophilic mice/rats was associated with the overexpression of MMP and a high ECM turnover [45]. The synovial vessels were distorted, large, elongated and thickened due to the proliferation of VSMC, and fibrotic myofibroblasts were highly present in the remodeled arteries of intraarticular soft tissues in both humans and mice with hemophilia [44]. They have features of contractile smooth muscle cells, and they are substantially involved in vascular remodeling. Angiogenesis led to increased vascularization, and it should be emphasized that hypervascularity was present both in the joints with hemarthrosis and in the otherwise unaffected ones. Thus, it can be concluded that bleeding in a joint causes systemic angiogenic stimuli. The local activation of the inflammation pathways and the intense synthesis of proinflammatory cytokines further contribute to abnormal vascular structures and microvascular flow in the affected joints [46,47].

In the human studies, increased vascularity was identified, and this reflects the intense local vascular remodeling [42]. Barnes et al. assessed the microvascular flow of large joints in hemophiliacs by ultrasound, and they showed increased vascularity in the intraarticular soft tissue. There was a correlation with the BP values as well: the higher the joint tissue perfusion was, the greater the risk that the patient was hypertensive [42]. This study hypothesized that recurrent joint bleedings and vascular remodeling may be accompanied by the overexpression of local and/or systemic mediators of angiogenesis and inflammation, which in turn may lead to abnormal BP control. Although the cross-talk paths between HTN and hemophilic arthropathy and the magnitude of this phenomenon has remained unknown, it was shown that vascular remodeling in hemophilic joints contributes to a greater risk of HTN and an elevated BP [42].

### 2.6. Endothelial Dysfunction

The possible contribution of hemophilia-related endothelial dysfunction in the pathogenesis of cardiovascular disease was investigated in a few studies, mainly focusing on endothelium-dependent vasodilation. Brachial flow-mediated dilation (FMD) assessed the degree of vasodilatation which was determined by the release of nitric oxide from the endothelium. FMD was measured in hemophiliacs and controls, with and without obesity. Neither the presence of hemophilia nor the presence of obesity influenced the endothelium-dependent vasodilation, although the amount of atherosclerosis was more significant in the obese individuals when they were compared to the non-obese individuals [2]. Another study found that FMD was impaired in hemophiliacs when they were compared to the general population, regardless of the severity of the disease, while the atherosclerotic burden was similar [48]. Moreover, the FMD impairment was present in the hemophiliacs without cardiovascular risk factors, which highlighted the effect of viral infections on the endothelium function. Hemophiliacs with an active viral infection—HCV and/or HIV—had the worst FMD values.

**Figure 3:**
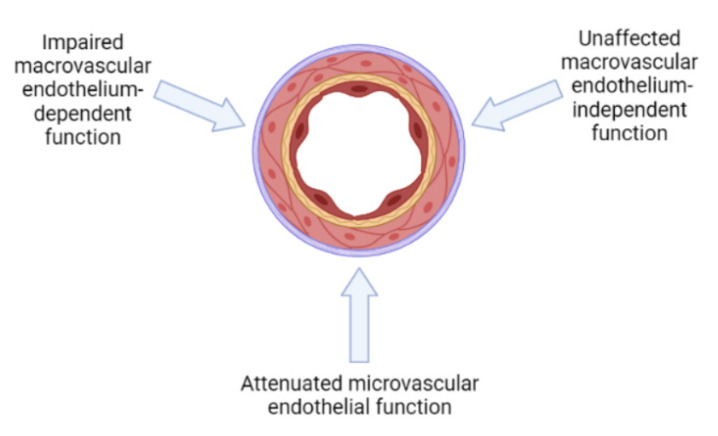
Endothelial function in patients with congenital hemophilia.

While previous studies assessed only the endothelium-dependent vasodilation and found contradictory results, Sun et al. investigated both the macrovascular and microvascular endothelial functions [49]. The study’s main findings were that hemophiliacs have a similar macrovascular endothelial function, but inferior microvascular endothelial function when they are compared to healthy controls. It should be emphasized that the controls were all men, who had been age- and cardiovascular risk-matched. These results are very important because the microvascular endothelial function is more strongly associated with the cardiovascular risk factors, and it predicts future cardiovascular events better than FMD can [50]. Since an impaired endothelial function is linked to HTN occurrence and progression, it could be one of the contributors to the higher prevalence of HTN in hemophiliacs than in the general population (Figure 3).

### 2.7. Viral Infections (HCV, HIV)

Extrahepatic conditions caused by an HCV infection must be considered because cardiovascular disease, CKD, obesity, dyslipidemia, insulin resistance and type 2 diabetes mellitus are among them. HCV causes glomerular and tubulointerstitial damage, which can progress to kidney failure. The most common kidney disease is membranoproliferative glomerulonephritis, which often leads to severe and difficult-to-control HTN. Individuals with a chronic HCV infection have a 1.7 times increased risk of developing type 2 diabetes, but they may also develop insulin resistance without diabetes [51]. Moreover, HCV is responsible for metabolic disturbances in the liver and adipose tissue, which determines an increased prevalence of obesity and metabolic syndrome [52]. HCV is linked to cardiovascular disease by multiple and complex mechanisms. Chronic inflammation leads to endothelial dysfunction and contributes to the development of atherosclerosis. This effect is potentiated by the increase in the prevalence of other cardiovascular risk factors, and also, a result of a chronic HCV infection. It was shown that chronic HCV infections are associated with increased peripheral arterial stiffness [53], and they are a risk factor for HTN. The more extensive the liver fibrosis was, the more frequent the HTN was [54].

Individuals living with HIV have an increased risk of HTN due to systemic chronic inflammation, renal disease and blood vessel damage resulting from the long-term exposure to the virus and antiretroviral therapy. An HIV infection itself is responsible for endothelial dysfunction that leads to increased arterial stiffness and premature atherosclerosis, even in patients with undetectable viremia [55]. Up to 30% of the HIV-infected patients develop renal complications, sometimes progressing to CKD. CKD is the result of kidney damage due to HIV-associated nephropathy, immune-complex glomerulonephritis, thrombotic microangiopathy, vasculitis, coinfection with HCV and antiretroviral therapy-induced renal toxicity [56].

Many hemophiliacs have been infected with HIV and/or HCV through plasma-derived clotting factors before blood product testing became mandatory [32]. These infections became frequent comorbidities. Among 3422 hemophiliacs, Kulkarni et al. found a prevalence of HCV and HIV infections in 31.9% and 24.2% of them, respectively [57]. In a cohort of 709 North-European hemophiliacs, the overall prevalence of HCV and HIV infections was 32% and 11%, respectively. When one is considering only those with severe hemophilia, the prevalence increased to 47% and 22%, respectively [30]. In a cohort of 185 hemophiliacs older than 60, the prevalence of HCV and HIV was 45% and 4%, respectively [29]. When considering only the patients with a severe disease, the prevalence increased to 85% and 11%, respectively. In a Taiwanese study, among 1054 hemophiliacs, 14.42% of them had an HCV infection and 2.85% of them had an HIV infection, which was significantly higher than 0.71% and 0.11% in the general population, respectively. [22].

The contribution of HCV and HIV to the pathogenesis of HTN in hemophiliacs was evaluated in several studies, and it was shown that HCV/HIV infections were associated with higher BP values and the prevalence of HTN [27]. HCV infections contribute to higher BP values especially in hemophiliacs over 30 years old [58]. Barnes et al. identified in a large cohort of 691 hemophiliacs that the risk of HTN was increased by an HIV infection [16]. This association was confirmed by a study of 60 HIV-infected hemophiliacs, which reported a prevalence of HTN in 64% of them, which is almost double that of the general population at 33% [59].

### 2.8. Hemophilia Treatment

Since its introduction in the 1970s, the hemophilia treatment has steadily increased the longevity and improved the quality of life in hemophiliacs. Fresh frozen plasma, frozen cryoprecipitate and plasma-derived concentrates were initially available. However, the donor blood was not tested, and as a result, many hemophiliacs were infected with HIV and/or HCV through plasma-derived clotting factor products. Immunogenicity was also important, as approximately 20% of the patients developed inhibitors [60].

Since then, the treatment has undergone great changes. Not only are the modern blood products tested and free of viral contamination, but the new drugs have been developed and implemented into current practice. Activated factors produced by recombinant DNA technology and factor-free replacement therapy have emerged, and the treatment is now heading towards gene therapy. Thus, the burden of iatrogenic comorbidities decreased. The concern about the viral safety of the recombinant coagulation factors was only temporary, which were related to first-generation factor VIII concentrates, and it was overcome. However, the high risk of inhibitor development remains a major concern even in the era of immunotolerant induction protocols [61]. Bypassing agents such as recombinant activated factor VII and plasma-derived activated prothrombin complex concentrate have long been the only options for patients with inhibitors, but their use was burdened by the unpredictability of the effect.

The monoclonal antibody emicizumab is the first factor-free replacement antihemophilic drug, and it has tremendous benefits in preventing bleeding in both of the categories of HA patients, with or without inhibitors. It is a new and modern treatment that aims to overcome all of the shortcomings of the previous therapies. The serious side effects are only related to the rare combination with activated prothrombin complex concentrate in the treatment of breakthrough bleeding [62].

Of interest are also the antifibrinolytics, such as tranexamic acid and ε-aminocaproic acid, which are used to control bleeding in different clinical scenarios. Although they are useful for controlling oral mucosal bleeds, they are currently contraindicated as initial therapies for upper urinary tract hematuria because clots can form and cause obstructive uropathy [34,35].

### 2.9. Studies Sowing Low Prevalence of HTN

The lowest prevalence of HTN in hemophiliacs (of 3.8%) was reported by Kulkarni et al., and their study reflects the large number of young patients who were included, wherein 64.5% of them were younger than 30 years and 79.4% of them were younger than 40 years. At these ages, the prevalence of HTN is generally very low [57]. Even more intriguing results were published by Seaman et al. who assessed the prevalence of HTN in a large US national database [63]. The prevalence of HTN in hemophiliacs was significantly lower than that of the general population. In this cohort of hemophiliacs there were fewer instances of obesity, hyperlipidemia, diabetes and CKD, and more instances of HCV, HIV and hematuria than there were in the general population.

A low prevalence of HTN was found in Swedish hemophiliacs [31]. Although the prevalence of NTH was higher in the hemophiliacs than it was in the general population, it was still significantly lower when it was compared to that which has been described so far. Because the Swedish health system has been implementing substitution treatment in patients with severe hemophilia for decades, it was initially thought that the low prevalence of HTN is partly determined by this practice. However, the insignificant difference in the prevalence of HTN in patients with severe vs. non-severe hemophilia did not support this hypothesis.

Furthermore, a European study that included hemophiliacs over 60 years of age found a lower prevalence of HTN when they were compared to the general population [29]. The patients with a severe disease had a slightly higher prevalence of HTN when they were compared to those with a non-severe disease. Although a clear explanation of this phenomenon was not provided, the higher incidences of hematuria, HCV, HIV and diabetes were noted in those with severe hemophilia when they were compared to those with the non-severe form of the disease.

Similar results were provided by the large Taiwanese study. Although the prevalence of HTN was low in hemophiliacs, it was similar to that in the general population [22] (Table 1).

### 2.10. Obesity, Diabetes Mellitus and Dyslipidemia in Hemophiliacs

There is a high amount of evidence that obesity, insulin resistance and dyslipidemia increase the BP in susceptible individuals in the general population. Therefore, their contribution to HTN and to cardiovascular risk was of interest to hemophiliacs as well. In individuals with hemophilia, obesity is the consequence of sedentary behavior, and several major determinants are recognized. One is hemarthrosis which leads to chronic hemophilic arthropathy and pain. Another is the fear of trauma and bleeding that leads to limited mobility and lack of proper physical exercise. Along with obesity, other metabolic disorders are common in hemophiliacs, such as dyslipidemia and diabetes, which are also as a consequence of a sedentary lifestyle.

Body mass index (BMI) and a prevalence of overweight/obesity were assessed in many studies, but the results varied widely and were often contradictory. It was reported that the prevalence of overweight/obesity in European and North-American hemophiliacs reaches 31%. While some studies have reported that there is a lower BMI and prevalence of obesity in patients with hemophilia when they are compared to the general population [10,30,58], others have found that there is a similar [23,73] or higher prevalence [21]. A study on Northern European hemophiliacs found an increasing prevalence of overweight and a doubling of obesity over a 10-year period [73]. It was highlighted that hypertensive patients usually have higher BMI than those with normal BP values [11,15]. HTN was 1.8 times more prevalent in overweight or obese hemophiliacs than in those of a normal weight [10].

Many studies reported that hemophiliacs have a better lipid profile than the men in the general population do [9,23,30,42,67]. Even in hemophiliacs aged over 65, hypercholesterolemia is rare [12]. These results are partially explained by the presence of liver damage due to a chronic HCV infection, which is highly prevalent in older hemophiliacs. Of note, HIV-infected hemophiliacs had more incidences of hypertriglyceridemia (60%) than the general population did [59]. Dyslipidemia was found in 22.4% of Canadian hemophiliacs [18]. In the Brazilian study, 41% of the hemophiliacs had hypercholesterolemia, 30% of them had low HDL-cholesterol and 18% of them had hypertriglyceridemia [74].

The prevalence of diabetes in hemophiliacs varies widely across studies, from a lower prevalence compared to the general population [58] to a similar [30] or a higher one [65]. The prevalence of it was 9.2% in Asians [21], 6.1% in Europeans [30], 10.5% in Canadians [18] and 16% in Brazilians [19]. One study found that one in four hemophiliacs had hyperglycemia [67]. Moreover, HIV-infected hemophiliacs had more incidences of diabetes (24%) than the general population did (6%) [59]. Recently, the presence of diabetes was related to a high risk of HTN [16]. In a large cohort of 691 hemophiliacs, Barnes et al. identified that the risk of HTN is severely increased by diabetes and to a lesser extent by HIV infection [16].

## 3. Discussion

The life expectancy of hemophiliacs has increased due to the wide availability of replacement therapies, and the comorbidities related to aging have become present. By analyzing more than 10,000 hospitalizations of hemophiliacs, it was found that HTN ranked first among the comorbidities, with it being identified in 33.4% of the admitted patients [72]. One study showed that among the cardiovascular diseases, HTN was the most common cause of an emergency department presentation [75]. Since HTN is an established major cardiovascular risk factor and it is strongly associated with hemorrhagic stroke, the study of HTN in hemophiliacs is of utmost importance.

The vast majority of studies have shown an increased prevalence of HTN in hemophiliacs, which is higher than that of the general population. It was also highlighted that the prevalence of HTN starts to increase at younger ages than it does in the general population. Therefore, BP measurements should be part of standard care in hemophilia patients and since one in four hemophiliacs aged 30–40 years old had an HTN, the screening should start early in life, at the age of 30 [10]. Another element that supports routine BP monitoring is that hemophiliacs do not seem to respond to antihypertensive treatment in the same way as the general population [58]. Assuming that there was similar compliance to treatment, the hemophiliacs had a BP profile with higher BP values, both systolic and diastolic, when they were compared to the general population. Of special interest is that the diastolic BP under treatment remained significantly higher in the hemophiliacs than it did in the treated controls, suggesting the greater stiffness of the vascular walls.

Some studies have reported an equal or lower prevalence of HTN in hemophiliacs than in the general population, and the main reason seems to be the studied population. A Swedish study found a low prevalence of HTN in hemophiliacs, at 19.7% [31]. Nonetheless, the prevalence was low in the general population as well, which resulted in an almost double prevalence of HTN in the hemophiliacs when they were compared to the general population. Thus, in this study, the low prevalence of HTN in hemophiliacs reflected the low prevalence of the disease in the Swedish population. The study of Seaman et al. [63] was the first to report a lower prevalence of HTN in hemophiliacs who were compared to the general population. One explanation was that this study included hospitalized patients, a cohort that might not be representative of the entire population of hemophiliacs. Another explanation was that the reference studies were retrospective or cross-sectional and potentially subject to inherent biases.

The influence of major cardiovascular risk factors on the BP profile was assessed in several studies. Their weight, lipid profile and fasting glucose values were frequently abnormal [24,25]. One study reported that one in two patients was obese or overweight, and one in four had a metabolic syndrome [24]. The clustering of cardiovascular risk factors was identified in several studies. The negative vascular effect of HTN was enhanced, and HTN was aggravated, which usually leads to an increased risk of coronary and cerebrovascular events. The lifetime prevalence of the cardiovascular disease among hemophiliacs reaches even 20% [69]. In a North-American study, 39% of the hemophiliacs had two or more cardiovascular risk factors. In the ARCHER study, the cardiovascular risk factors were common, with three out of four hemophiliacs having at least one risk factor [18]. Of 294 hemophiliacs, 30.0%, 21.8%, 9.9% and 5.8% of them had one, two, three, or at least four risk factors, respectively. In a cohort of 469 hemophiliacs, both the systolic and diastolic blood pressures were higher in the hemophiliacs than they were in the controls, even after adjusting for age, BMI, diabetes, cholesterol and renal function [58]. Thus, traditional risk factors alone are unlikely to explain the higher prevalence of HTN in hemophiliacs [15], although the interactions between these risk factors and hemophilia status cannot be entirely ruled out.

The role of hemophilia-specific factors has been addressed in many studies, but the contribution of the type of hemophilia, its severity or the presence of inhibitors is not supported by consistent results across studies. The bidirectional relationship between renal dysfunction and HTN as well as that between endothelial dysfunction and HTN gathered much evidence that supports their deep involvement in the pathogenesis of HTN in individuals from the general population. Therefore, their potential role as risk factors for NTH in hemophiliacs has been investigated with great interest. Urinary tract bleedings, which are clinically expressed as hematuria, are one of the most common manifestations of hemophilia after hemarthrosis. However, the correlation between hematuria and the incidence and severity of renal dysfunction varied across studies, which did not allow a definitive conclusion to be drawn [11,76]. Of note, HTN acts as a trigger for hematuria, closing a pathogenic loop. The high prevalence of hematuria, HIV and HCV, with all of their direct renal or systemically mediated consequences, represents one of the possible substrates of HTN in hemophiliacs [11,36,39]. New concepts regarding endothelial dysfunction in hemophiliacs are gaining evidence, and its possible contribution to the pathogenesis of HTN is currently under intense debate [2,48,49]. Even the newest association between HTN and hemophilic arthropathy is taking shape, emphasizing the local and systemic role of mediators of inflammation and angiogenesis. Originating or triggering at the level of the joint affected by hemarthrosis, these mediators seem to exert systemic vascular effects, but the mechanism is far from being elucidated [42,44,45,77].

HTN is a major contributor to cardiovascular risk, and the studies showed that hemophiliacs seem to have a more unfavorable cardiovascular profile than the general population does, especially due to the increased prevalence of HTN. Hemophiliacs have a 20 to 50 times greater risk of intracranial hemorrhage (ICH) than the general male population does, resulting in a mortality of up to 21.9%. In children and adolescents, 90% of the ICHs are associated with a severe disease, but in those over 50, half of all of the ICH cases occur in patients with the mild and moderate diseases [70]. HTN, and especially uncontrolled one, is an important contributor. This highlights that age or high blood pressure play crucial roles as the hemophiliac ages. The Italian EMO.REC registry found that of 31 hemophiliacs with ICH, 17 had HTN, of which 11 had a mild disease [78].

Given the accumulation of cardiovascular risk factors, we are currently facing an increase in the number of cases of coronary artery disease in hemophiliacs. It was shown that a higher prevalence of cardiovascular risk factors in hemophiliacs, such as HTN, reflects in a significantly higher prevalence of coronary artery disease. Of 2506 American hemophiliacs, 22.6% of them had HTN, 15.9% of them hyperlipidemia and 10.7% of them coronary artery disease compared to 15.5%, 11.9% and 5.8% of the age-matched males in the general population, respectively [17]. Of 35 North-American hemophiliacs with no prior cardiovascular event, 54.3% of them had an estimated 10-year risk which was above 10% [68]. A Netherlands study reported that 12% of hemophiliacs were in the 10-year cardiovascular mortality risk > 10% group [67], a percentage that is comparable to the one that has been found in the general population. The main contributor to the increased risk was HTN, which is more prevalent in hemophiliacs than in the general male population. In the Brazilian cohort, 39% of the hemophiliacs were in the high-risk category [19]. An additional 35% of the hemophiliacs were in the moderate-risk category. All these data show that there is a high cardiovascular burden in hemophiliacs and the importance of the correct management of HTN.

Our study has several limitations. Firstly, hemophilia is a rare disease and the data that were analyzed mainly came from small studies which were published by treatment centers dedicated to hemophiliacs. Secondly, there is no consistency across the studies regarding the analyzed parameters, and moreover, the results are sometimes divergent, which increases the difficulty of interpreting the results and reaching a conclusion.

## 4. Conclusions

Aging hemophiliacs face cardiovascular disease. Arterial hypertension is much more prevalent, and it determines a more unfavorable cardiovascular profile in hemophiliacs than it does in the general population. The mechanism of HTN in hemophiliacs is complex and multifactorial, and it has determinants in direct relation to the disease. Although great progress has been made in the elucidation of the main pathogenic links, there are still elements that require further research. Among the existing evidence, our study is valuable because, as far as we know, it is the most comprehensive and systematized analysis of the determinants of HTN in hemophiliacs. Moreover, it increases the degree of awareness regarding the peculiarities of HTN in hemophiliacs, and we hope it will enhance its management.

## Figures and Tables

**Table 1:** Prevalence of HTN in patients with congenital hemophilia.

Author, Year	Region	No. Patients, HA (%)	Population, Mean Age (Years)	Prevalence of HTN in Hemophiliacs vs. Comparator ^b^ (%)
Rosendaal et al., 1990, [9]	Europe	95 92% HA	Adults 38 years	23.15%
Bilora et al., 1999, [64]	Europe	76 HA and vWD	Adults 58.2 years	28.9%
Kulkarni et al., 2005, [57]	North-America	3422 79.3% HA	All ages 20.8% ≥ 40 years	3.8%
Walsh et al., 2008, [65]	North-America	47 100% HA	46 years	29% vs. 18%
Miesbach et al., 2009, [14]	Europe	185 92% HA	≥60 years 69 years	55%
Siboni et al., 2009, [12]	Europe	39 85% HA	≥65 years	Higher (*p* = 0.035)
Foley et al., 2010, [66]	North-America	14 100% HA	40 years	28.6% vs. 34.2%
Biere- Rafi et al., 2011, [67]	Europe	100 89% HA	47 years	51% vs. 37.5% (*p* = 0.03)
Lim et al., 2011, [68]	North-America	58 70.7% HA	55.7 years	65.5% vs. 53.2% (*p* = 0.066)
Sharathkumar et al., 2011, [69]	North-America	185 55.1% HA	52.5 years	68.1%
Von Mackensen et al., 2011, [13]	Europe	39 85% HA	≥65 years	71.8% vs. 44.2% (*p* = 0.010)
Fransen van de Putte et al., 2012, [10]	Europe	701 patients 84% HA	≥30 years 49.8 years	49% vs. 40%
von Drygalski et al., 2013, [15]	North-America	458 79% HA	≥18 years 40 years	49.1% vs. 31.7% (*p* < 0.0001)
Pocoski et al., 2014, [17]	North-America	2506 100% HA	All ages 39% ≥ 40 years	22.6% vs. 15.5% all ages (*p* < 0.001)
Minuk et al., 2015, [18]	North-America	294 75.5% HA	54 years	31.3%
Wang et al., 2015, [22]	Asia	1054 84% HA	All ages 23.2% ≥ 40 years	10.63% vs. 8.94%
Holme et al., Berger et al., 2016, [11,70]	Europe	532 87.8% HA	52 years	45.2% 52.0% vs. 41.7%; (*p* = 0.03 for 50–59 years) *p* = 0.48 for 40–49 years *p* = 0.90 for 60–69 years *p* = 0.29 for 70–79 years
Barnes et al., 2016, [58]	North-America	469 79.1% HA	≥18 years	Higher
Amoozgar et al., 2017, [71]	Asia	50 84% HA	29.1 years	Higher 10%—systolic HTN 14%—diastolic HTN
Seaman et al., 2017, [63]	North-America	3607 ^a^ NR	49.32 years	39.47% vs. 56.3%
Miesbach et al., 2017, [29]	Europe	185 92% HA	≥60 years 69 years	45.8% vs. 51.8% (*p* = 0.246, 60–69 years) 52.5% vs. 64.9% (*p* = 0.045, 70–79 years)
Limjoco et al., 2018, [25]	North-America	43 NR	≤20 years 12 years	28%—(pre) hypertension
Nagao et al., 2019, [23]	Asia	711 82% HA	≥30 years 45 years	43.7% (*p* = 0.107)
Lövdahl et al., 2019, [31]	Europe	1431 981	born before 2009 born in 1978 or earlier	13.7% vs. 7.7% 19.7% vs. 11.2% (*p* < 0.001)
Babaeva et al., 2021, [39]	Europe	93 92.5% HA	≥18 years	27.9%
Camelo et al., 2021, [19]	South-America	82 83% HA	≥30 years 43 years	60%
Aydin et al., 2022, [20]	Asia	26 100% HA	44 years	19%
Day et al., 2022, [72]	North-America	8623 ^a^ NR	Adults 54 years	33.4%
Vithanage et al., 2022, [21]	Asia	109 84.4% HA	≥18 years 36 years	27.5%
Ter-Zakarian et al., 2022, [27]	North-America	691 77% HA	≥18 years 39 years	Higher

^a^ = hospitalizations; ^b^ = since not all studies compared the prevalence of HTN in hemophiliacs with that in the general population, only existing data were included; HTN = hypertension; HA = hemophilia A; NR = not reported.

## Data Availability

Not applicable.
